# Relationship between Thermal Diffusivity and Mechanical Properties of Wood

**DOI:** 10.3390/ma15020632

**Published:** 2022-01-14

**Authors:** Yuri I. Golovin, Alexander I. Tyurin, Dmitry Yu. Golovin, Alexander A. Samodurov, Sergey M. Matveev, Maria A. Yunack, Inna A. Vasyukova, Olga V. Zakharova, Vyacheslav V. Rodaev, Alexander A. Gusev

**Affiliations:** 1 Institute “Nanotechnology and Nanomaterials”, G.R. Derzhavin Tambov State University, 392000 Tambov, Russia; yugolovin@yandex.ru (Y.I.G.); tyurinalexander@yandex.ru (A.I.T.); tarlin@yandex.ru (D.Y.G.); samsasha@yandex.ru (A.A.S.); mascha150383@mail.ru (M.A.Y.); vasyukovaia@gmail.com (I.A.V.); olgazakharova1@mail.ru (O.V.Z.); rodaev1980@mail.ru (V.V.R.); 2 Department of Chemical Enzymology, School of Chemistry, Lomonosov Moscow State University, 119991 Moscow, Russia; 3 Research Educational Center «Sustainable Development of the Forest Complex», Voronezh State Forestry University Named after G.F. Morozov, 394087 Voronezh, Russia; lisovod@bk.ru; 4 Department of Functional Nanosystems and High-Temperature Materials, National University of Science and Technology “MISIS”, 119991 Moscow, Russia

**Keywords:** thermal diffusivity, dynamic thermal imaging, mechanical properties, Brinell hardness, microhardness, Young’s modulus

## Abstract

This paper describes an experimental study of the relationships between thermal diffusivity and mechanical characteristics including Brinell hardness, microhardness, and Young’s modulus of common pine (*Pinus sylvestris* L.), pedunculate oak (*Quercus robur* L.), and small-leaf lime (*Tilia cordata* Mill.) wood. A dependence of Brinell hardness and thermal diffusivity tensor components upon humidity for common pine wood is found. The results of the measurement of Brinell hardness, microhardness, Young’s modulus, and main components of thermal diffusivity tensor for three perpendicular cuts are found to be correlated. It is shown that the mechanical properties correlate better with the ratio of longitude to transversal thermal diffusivity coefficients than with the respective individual absolute values. The mechanical characteristics with the highest correlation with the abovementioned ratio are found to be the ratio of Young’s moduli in longitude and transversal directions. Our technique allows a comparative express assessment of wood mechanical properties by means of a contactless non-destructive measurement of its thermal properties using dynamic thermal imaging instead of laborious and material-consuming destructive mechanical tests.

## 1. Introduction

It is well known that some mechanical properties of materials can be linked with simple relations due to their similar dependence on material nano- and microstructure. For instance, the hardness *H* of ductile metals depends upon the yield stress σ*_y_*, as *H* ≈ 3σ*_y_*, while for hard and superhard metals and alloys, it depends upon the shear modulus *G*, as *H* ≈ 0.15 *G* [1]. There is reason to believe that similar relations could link mechanical and thermophysical properties (TPP) of materials belonging to the same type. In particular, thermal conductivity λ and diffusivity *a* [2,3,4] of wood and other anisotropic composite materials depend on density, porosity, humidity [5,6,7], and specifics of interconnections between microstructural units of the material as hardness, Young’s modulus, and strength do [8,9,10]. Once revealed, such relations could allow switching from laborious and material extensively destructive mechanical tests to non-destructive measurements of TPP using express methods, as described in [11,12,13]. Typically, they require around 1 min per measurement and do not demand for cutting samples of any specific geometry. Such approach could be used to estimate relative mechanical properties, sorting, and grading of materials and products made of wood, fiber-reinforced composites etc.

There is a lot of information concerning the mechanical and thermal properties of natural [6,8,10] and modified [14] wood, wood-based layered materials [3,15,16], as well as composites reinforced with artificial [5,9] and natural organic fibers [17,18,19]. For example, it is found that the thermal conductivity of bamboo measured by the classical stationary method along the fibers λ*_l_* exceeds the transversal one λ*_n_* by nearly 2 times [20], and both increase linearly with material density. In [21], the mapping of the thermal conductivity of bamboo cellular structure at the nano- and microscale was carried out by means of scanning thermal microscopy. It provided information about the relative thermal conductivity of different layers of the cell wall and revealed that the relative thermal conductivity λ* significantly depended upon the angle between the microfibers forming the specific layer of cell wall and cell long axis. Thermal conductivity and diffusivity measurements of birch wood in longitude and transverse directions were reported and discussed in [4]. Instances of correlations between thermal conductivity and stiffness of specific layers of the cell wall can be implicitly found in [22].

However, the mechanical and thermal properties are measured in separate tests executed on different samples and are not related to each other, except for several papers that use thermal non-destructive testing to estimate structural damage of wood [23,24,25], defects [26,27], porosity [28], and anisotropy [29,30] of composites and their possible impact on material mechanical properties. In [31], a micromechanical model for the prediction of effective thermal conductivity in two- and three-phase composites is proposed.

The goal of the current paper was the study of correlations between Brinell hardness HBW, microhardness *H*, Young’s modulus *E*, and thermal diffusivity tensor *a_ij_* components in common pine (*Pinus sylvestris* L.), pedunculate oak (*Quercus robur* L.), and small-leaf lime (*Tilia cordata* Mill.) wood. The samples had no visible defects or mechanical damage and demonstrated significant anisotropy in mechanical properties and TPP.

## 2. Materials and Methods

The studied samples were taken at sanitary felling in natural forests of the Central Black Earth Region of Russia in June–September 2021. Common pine (*Pinus sylvestris* L.) samples were from 110–125-year-old trees originating from Vernadsky forest district of Tsna woodland in Tambov region, pedunculate oak samples (*Quercus robur* L.) were from the trees that were 90–110 years old, and small-leaf lime (*Tilia cordata* Mill.) samples were from the trees that were 70–90 years old originated from an educational–experimental plot on the right bank forest district of Voronezh State Forestry University, named after G.F. Morozov. The age of the trees was determined by annual rings counting on a log crosscut.

**Figure 1:**
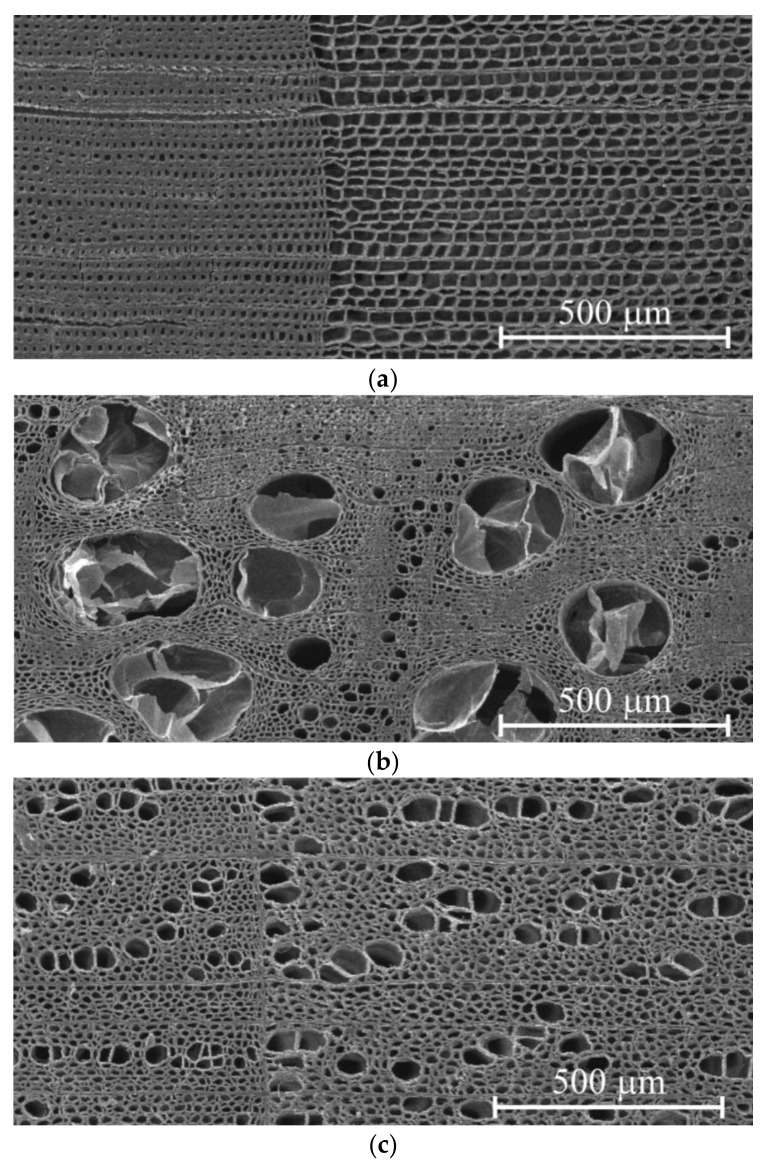
SEM images of crosscuts of common pine (**a**), pedunculate oak (**b**), and small-leaf lime (**c**) wood. The borders of growth rings are vertical and can be identified by an abrupt change of cells and a tracheid morphology.

Sample blocks with dimensions of 30 × 30 × 100 mm^3^ were cut from log crosscuts and dried to 10–12% humidity in a timber-drying kiln at 75 °C for 24 to 72 h. Sample surface was processed mechanically with a Buehler grinding-and-polishing machine (Buehler Inc., Lake Bluff, IL, USA) using abrasives with successively diminishing grain size. Polished surface roughness *R_a_* was determined using a di Innova (Veeco Instruments Inc., Santa Barbara, CA, USA) scanning probe microscope. A typical *R_a_* value for pine and lime samples was around 250–300 nm, while it was 140–180 nm for oak samples. Samples microstructure was studied using a Tescan Vega 3 (Tescan Orsay Holding, Brno, Czech Republic) scanning electron microscope. Surfaces for electron microscopy were prepared by cutting away thin layers from the polished surface using an ultramicrotome. Typical images of annual growth rings and cellular structure of the studied samples are presented in Figure 1. As can be seen, the typical grain size was 30–40 μm.

A nanotester Triboindenter TI-950 (Hysitron Inc., Minneapolis, MN, USA) was used for mapping the mechanical properties of the samples under study. It is a precision nano-/micromechanical testing machine that records load–strain diagrams with ~50 nN resolution in the load *P* channel and 0.5 nm resolution in the displacement *h* channel while impressing a sharp triangle-piramidal Berkovich indenter with a tip curvature around 20–50 nm into the tested surface. Its software and three-axis stage allowed presetting the loading–unloading rates during a working cycle up to ~1000 indentation positions, so that further mapping of the mechanical properties did not require operator control. Raw data were processed according to the Oliver–Pharr method [32,33,34] used in ISO 14577 [35] to obtain *H* and *E* values.

To apply the nanoindentation technique to study soft biological materials characterized by inhomogeneity at various scale levels, the properties of anisotropy, and time dependencies, the choice of the testing protocols, including maximal load *P*_max_, duration of holding at maximal load, etc., becomes very important [36]. In studying the nano-/micromechanical properties of wood cell wall, *P*_max_ is usually chosen in the range of 0.1–1 mN [37,38,39,40,41], so that the lateral size of the indent is much less than the cell wall width (2–5 μm). Such measurements provide interesting information about the contribution of different layers to cell wall stiffness and hardness. However, they are far from providing macromechanical properties as long as the latter are affected by porosity, the composition of the growth ring of early and late wood, and so on. Besides, due to the similarity of cell walls’ microstructure, nanohardness *H_n_* variations within the same sample and even between different wood species are not large. For instance, the measurements of *H_n_* in various pine species give the following values: 440–490 MPa for *Pinus sylvestris* L. [37], 350–420 MPa for *Pinus massoniana* Lamb., as reported in [38], and 410–530 MPa [39] and 340–540 MPa for *Pinus taeda* [40].

The chosen maximal load *P*_max_ = 500 mN ensured that the lateral size of the deformation region (80–130 μm) exceeded 3–5 times the lateral size of the cells, so that the measured mechanical characteristics were averaged over 10–25 nearby cells including cell walls and lumens. The values of *E*_IT_ and *H*_IT_ measured as above could be considered as effective values for a certain layer of the wood. Each point on the figures resulted from averaging 5 to 10 individual indentations carried out in the same conditions at the same distance from the start of a certain growth ring.

It is well known that each annual growth ring of any tree in the temperate zone consists of early wood (EW) and late wood (LW) layers. The former is formed during spring and has low mean density and strength, while the latter is formed during summer and autumn and has a somewhat higher mean density and significantly higher mechanical characteristics. A deliberately chosen load of 500 mN and, hence, deformed region size, provided a lower dispersion of the measured *H* and *E* values compared to previously reported data [42] obtained at a much lower load *P*_max_ = 2 mN, while still allowing a reliable study of *E*_IT_ and *H*_IT_ distributions within individual growth rings and demanding a moderate number of indentations for it.

The macroscopic mechanical properties were measured according to the Brinell method by means of indentation of a ceramic ball with 12.7 mm diameter into the crosscut surface up to the depth around 1 mm, so that the deformed region encompassed several growth rings. It allowed comparing macroscopic and microscopic hardness measured at the same sample and table values of hardness for the wood species. Macroscopic Young’s modulus was measured by three-point bending of 20 × 30 × 350 mm^3^ samples. All macromechanical tests were carried out using an MTS 870 Landmark (Eden Prairie, MN, USA) testing machine.

To determine the dependency of Brinell hardness HB and thermal diffusivity *a* on wood humidity *w*, the samples were dried to *w*_0_ = 12% and then held in a moist environment for various times to alter the water content. After this exposure, sample humidity *w* was determined by mass change just before the main experiment.

**Figure 2:**
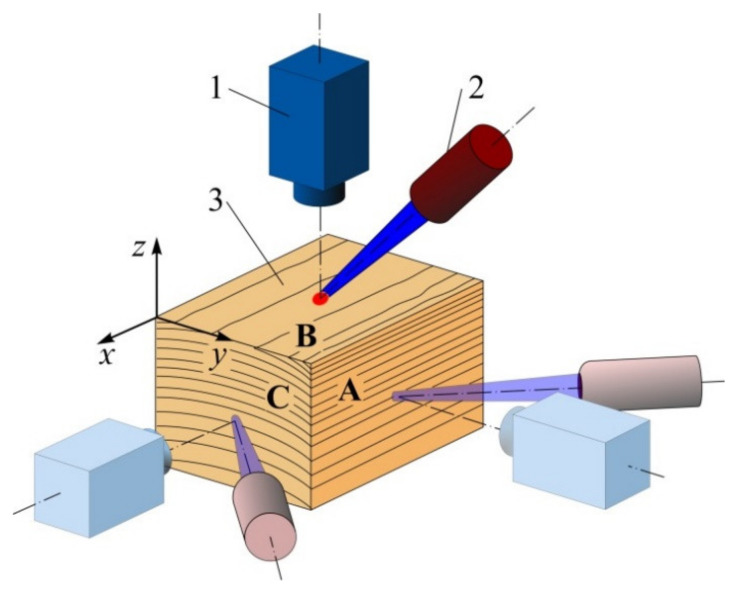
Thermal diffusivity measurement layout. 1—thermal camera, 2—laser, 3—wood sample, A—radial cut, B—tangential cut, C—crosscut.

The thermal diffusivity tensor components *a_ij_* = λ*_ij_*/ρ*C_p_*—where λ*_ij_* represents the thermal conductivity tensor components, ρ is the material density, *C_p_* is the specific thermal capacitance—were measured using our original non-destructive thermal imaging technique described in details in [11,12,13]. The idea at the basis is to perform a local stepped heating at small spots on the sample surface by a laser beam while continuously monitoring the surface temperature distribution with a thermal camera. Heat propagation in such setup is close to spherical symmetry in isotropic materials, while in h materials, the isothermal surfaces are close to three-axis ellipsoids with the axes fully determined by the main components of *a_ij_* tensor and the time elapsed since heating onset, provided that the distance to the heating center is at least several times higher than the heating beam radius. Therefore, the *a_ij_* values were determined by processing dynamic thermal images obtained on lateral, radial, and transverse crosscuts of wood samples, as shown at Figure 2.

**Figure 3:**
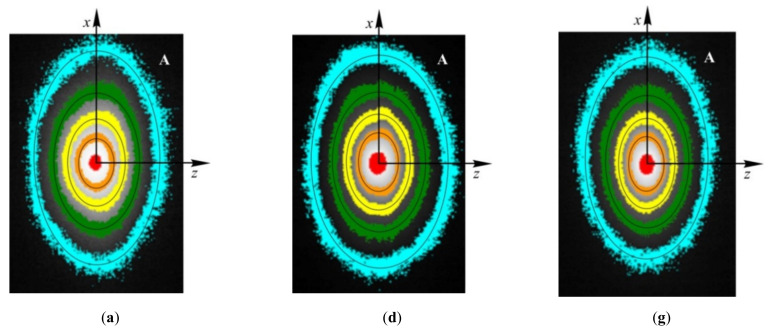

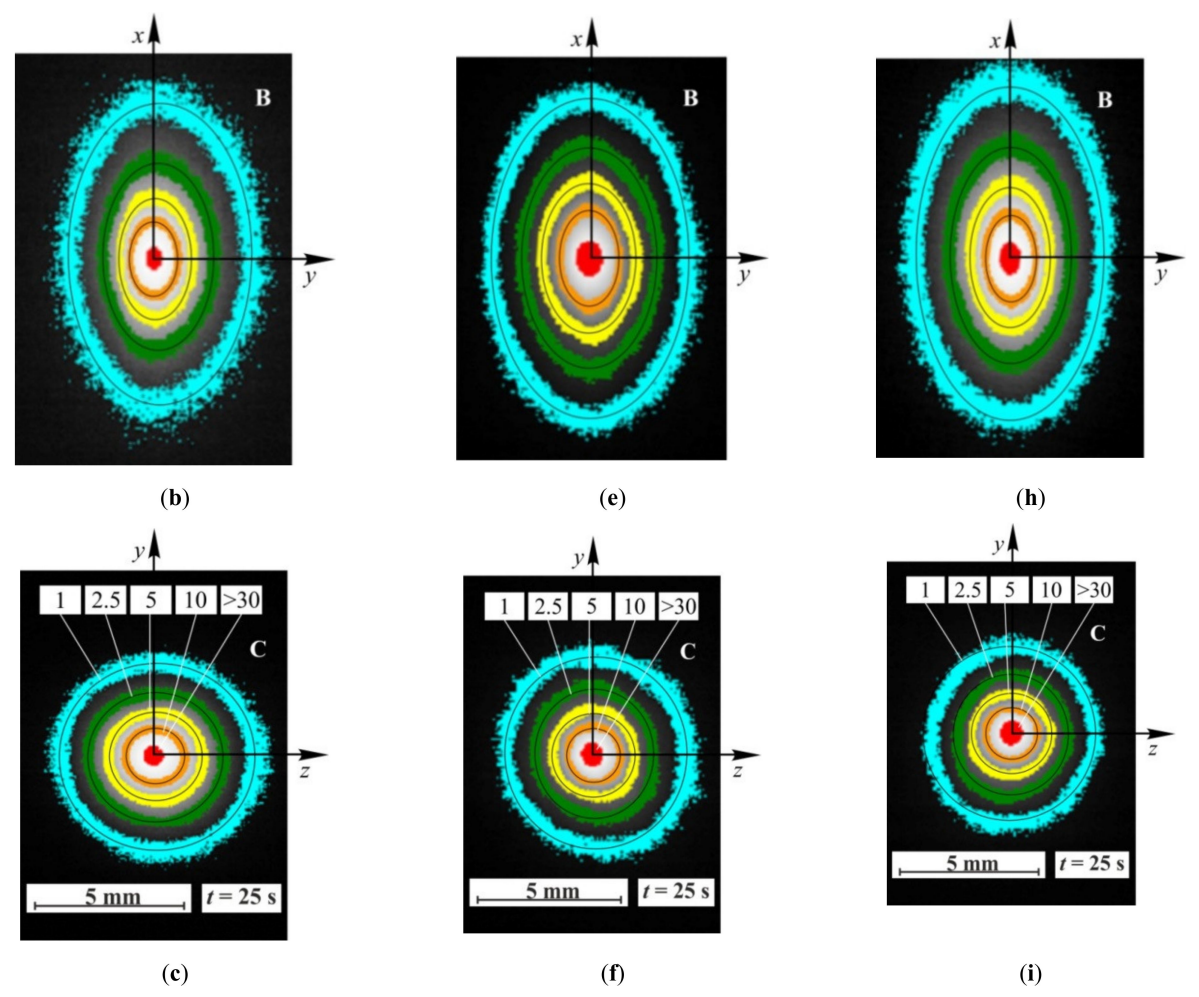
Thermal images for pine (**a**–**c**), oak (**d**–**f**), and lime (**g–i**) at A, B, and C faces: along the fibers, radial cut (**a**,**d**,**g**), along the fiber, tangential cut (**b**,**e**,**h**), and crosscut across the fibers (**c**,**f**,**i**). The number on the isotherms indicates the overheating temperatures above the ambient temperature: 1 ± 0.25, 2.5 ± 0.5, 5.0 ± 0.5, 10 ± 2, and >30 °C; *t*—time after heating onset.

The sample surface was heated locally by a focused light beam generated by a solid-state laser with diode excitation, LSR445CP-FC-10W (Lasever, Ningbo, China). The beam Gaussian radius *r*_0_ at the surface was 0.1–0.3 mm. The light pulse with duration of 30–60 s had constant power preset in the 0.3–1 W range. The dynamic thermal field on the sample surface was filmed by a thermal camera FLIR–A35sc (FLIR Systems, OR, USA) at the rate of 10–60 frames per second with the field of view of (10–20)*r*_0_ that was larger than the size of the region of significant temperature change. All three dimensions of the sample exceeded the field-of-view size significantly and did not affect the temperature distribution. Several instances of thermal images at three perpendicular faces with some processing are shown at Figure 3.

Every recorded frame was then processed according to the original models and algorithms described in [11,12,13,43,44]. In particular, each frame in a time interval such as 1–3 s after heating onset, with a pixel-by-pixel subtracted base frame obtained before heating, was used to determine the main axes of several tens of elliptical isotherms. This array of axes lengths depending on the temperature was approximated by a model function, so that each frame recorded at a known time yielded two values determined by three independent components of the *a_ij_* tensor. The values obtained for individual frames were then averaged in the above time interval, so that the method took into account the signals from many hundreds of thousands of pixels (~200 pixels per ellipse, ×~30 ellipses, ×~100 frames) to obtain two scalar values. This greatly reduced various types of noises, while the absolute calibration of temperature-to-signal transformation did not affect the results at all as long as it remained constant across the field of view. Each frame was processed individually, so that even the change of that calibration over time was insignificant. To compute real thermal diffusivity tensor components in the orthotropic case, at least two tests on perpendicular faces were required.

## 3. Results

**Figure 4:**
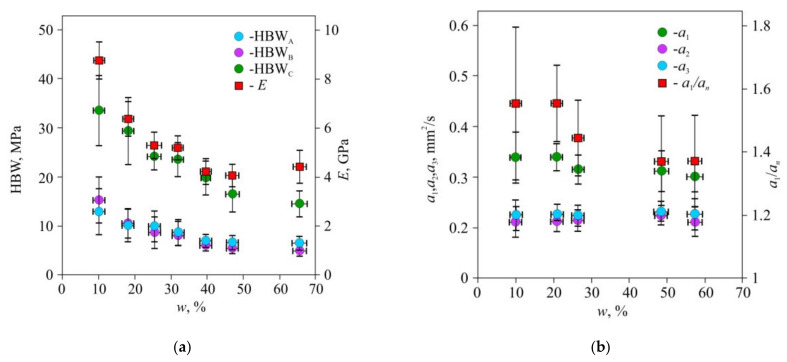
Dependency of Brinell hardness HBW and Young’s modulus *E* (**a**), thermal diffusivity coefficients *a_1_*, *a_2_*, *a_3_*, and *a_1_/a_n_* ratio (**b**) upon humidity w of pine wood. HBW_A_, HBW_B_, HBW_C_-Brinell hardness on radial, tangential, and cross-fiber cuts (A, B and C at Figure 2 accordingly). *E*—Young’s modulus across the fibers measured by three-point bending; *a*_1_, *a*_2_, *a*_3_—tangential, radial, and cross-fiber coefficients of thermal diffusivity; *a_n_* = (*a*_2_ + *a*_3_)/2.

In the first stage of the experiments, we studied Brinell hardness HBW, Young’s modulus *E* (Figure 4a), and the main components *a*_1_, *a*_2*,*_ and *a*_3_ of thermal diffusivity tensor (Figure 4b) dependencies in common pine wood on its humidity *w*. The absolute and relative values of *a*_1_, *a*_2_, and *a*_3_ are affected by *w* differently. The *a*_1_ component displayed significant dependence on *w* along the fibers, while across the fibers, the dependence of the components *a*_2_ and *a*_3_ upon *w* was weak, and they were statistically indistinguishable. Hence, it was reasonable to introduce the averaged value *a_n_* = (*a*_2_ + *a*_3_)/2 of thermal diffusivity normal to the fibers. The relative values *a*_l_/*a_n_* that characterize thermal diffusivity anisotropy were even more sensitive to humidity change than the absolute ones (Figure 4b).

**Figure 5:**
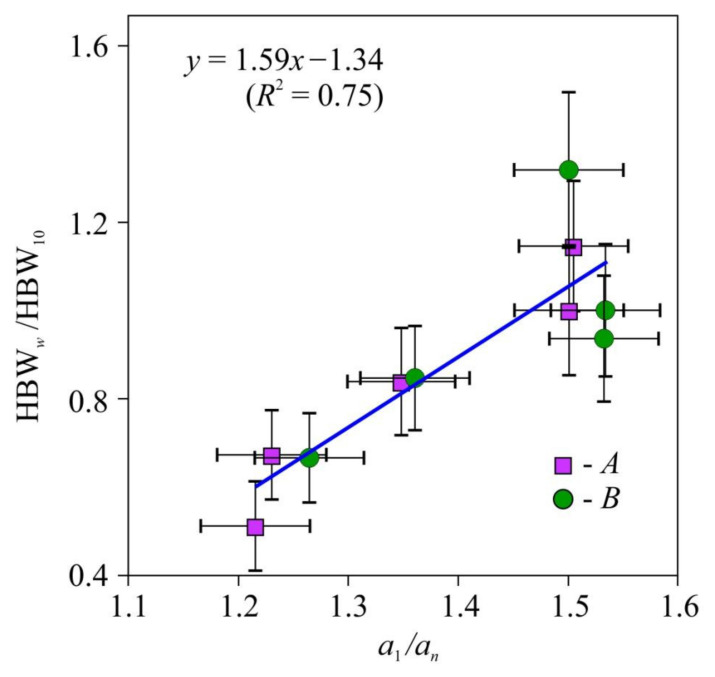
Dependence of the relative macrohardness HBW*_w_*/HBW_10_ of pine wood upon thermal diffusivity anisotropy—*a*_1_*/a_n_*. HBW—Brinell hardness (12.7 mm sphere diameter, 1 mm indentation depth), *a*_1_, *a_n_*—thermal diffusivity coefficients along and across the fibers. HBW*_w_/*HBW_10_—HBW values on the faces А and B, as shown in Figure 2 normalized to HBW at humidity *w* = 10% (HBW_10_).

The values of hardness HBW normalized to the hardness HBW_10_ at *w* =10% for the lateral and radial faces were statistically indistinguishable too (Figure 5); therefore, they were approximated as a single set by the following linear function (HBW/HBW_10_) = 1.59(*a*_l_/*a_n_*) − 1.34 with the coefficient of determination *R*^2^ = 0.75. The hardness measured on the face perpendicular to the fibers was found to be independent of tensor *a_ii_* components, so that it could be used for method and equipment calibration.

**Figure 6:**
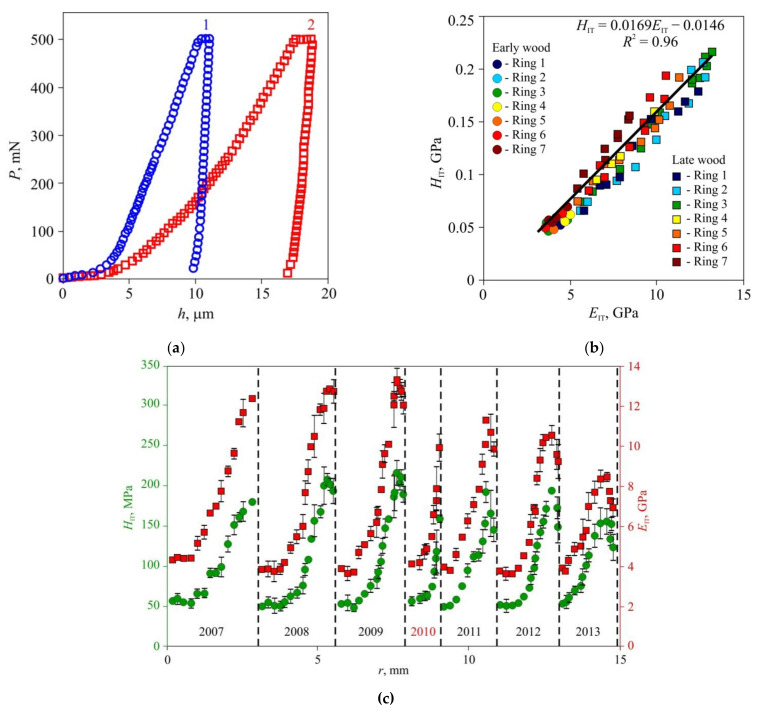
Effective microhardness *H*_IT_ and Young’s modulus *E*_IT_ in annual rings of pine wood, (**a**) Typical *P–h* diagrams for late (1) and early (2) wood; (**b**) dependence of hardness *H*_IT_ upon Young’s modulus *E*_IT_ for seven sequential growth rings; (**c**) spatial distribution of hardness *H*_IT_ and Young’s modulus *E*_IT_ across sequential growth rings *r*. The positions of ring boundaries are shown by dashed lines. The corresponding years for the growth rings are shown below the data points. An extraordinary dry year is indicated in red.

**Figure 7:**
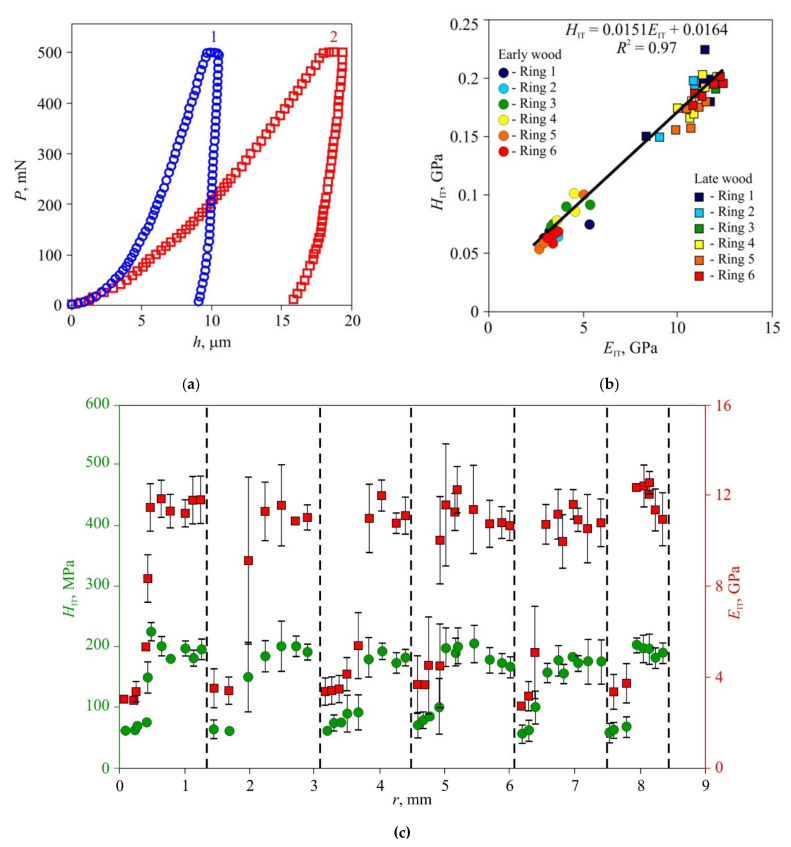
Effective microhardness *H*_IT_ and Young’s modulus *E*_IT_ in annual rings of oak wood, (**a**) Typical *P–h* diagrams for late (1) and early (2) wood; (**b**) dependence of hardness *H*_IT_ upon Young modulus *E*_IT_ for seven sequential growth rings; (**c**) spatial distribution of hardness *H*_IT_ and Young modulus *E*_IT_ across sequential growth rings *r*. The positions of ring boundaries are shown by dashed lines.

**Figure 8:**
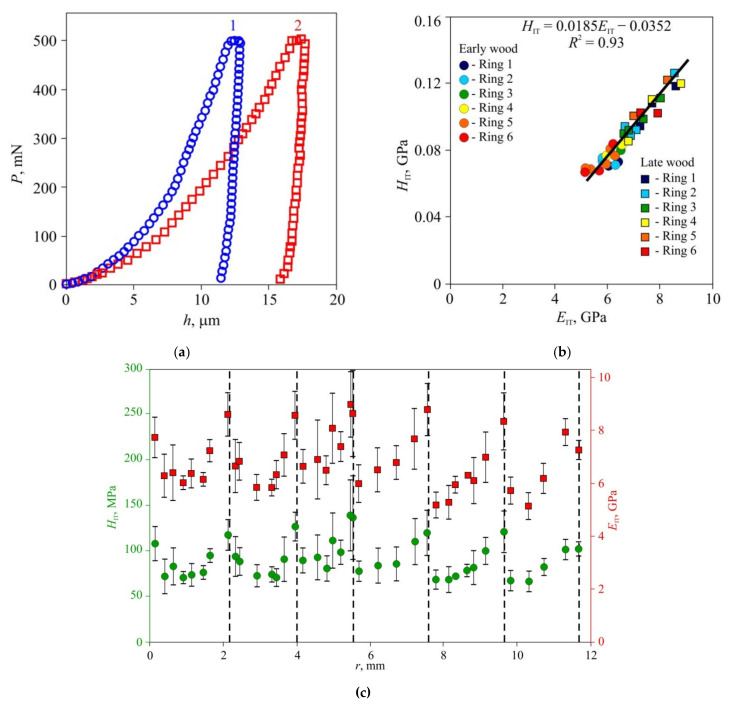
Effective microhardness *H*_IT_ and Young’s modulus *E*_IT_ in annual rings of lime wood, (**a**) Typical *P–h* diagrams for late (1) and early (2) wood; (**b**) dependence of hardness *H*_IT_ upon Young’s modulus *E*_IT_ for seven sequential growth rings; (**c**) spatial distribution of hardness *H*_IT_ and Young’s modulus *E*_IT_ across sequential growth rings *r*. The positions of ring boundaries are shown by dashed lines.

In the second stage of the experiments, microhardness and Young’s modulus in early and late wood layers, Brinell hardness HBW, and thermal diffusivity tensor main components were measured in dried wood of all three species (Figure 6, Figure 7 and Figure 8). Typical *P–h* curves recorded in NI for the three wood species are shown in Figure 6a, Figure 7a and Figure 8a. Wood of all three species displayed pronounced periodicity of local mechanical properties (Figure 6c, Figure 7c and Figure 8c). Locations of abrupt changes of *H*_IT_ and *E*_IT_ coincided with the boundaries of annual growth rings found using an optical method. Changes of *H*_IT_ and *E*_IT_ in transition regions between early and late wood in each growth ring were found to be gradual in pine and lime but stepped in oak. *H*_IT_ and *E*_IT_ were linked by a linear relation with the same slope *m* = 0.017 ± 0.002 for all three species (Figure 6b, Figure 7b and Figure 8b). In other words, the ductile ratio DR = *E*_IT_*/H*_IT_ for all studied species appeared to be around 60, which is typical of many wood species. For example, the DR for eucalyptus is between 54 and 68 [45], with an average of 61.

The values of *H*_IT_ and *E*_IT_ of the EW layer differed only slightly (within 10–15%) both within each ring and for different rings, despite the growth conditions differing significantly. For instance, in 2010, there was a severe drough, so that the width of the growth ring in pine wood was less than half its normal value; however, there was no discernible effect on *H*_IT_ and *E*_IT_ of EW. The lateral sizes of cells corresponding to different years had no visible difference too, but the wall thickness did differ (Figure 1). Therefore, the variation of the annual growth ring width was attributed to the different number of cells in the layer, while the size and mechanical properties of each cell remained almost the same. Effective hardness and Young’s modulus of the LW layers were several times higher than those of the EW layers due to higher cell wall thickness and lower lumen area.

**Figure 9:**
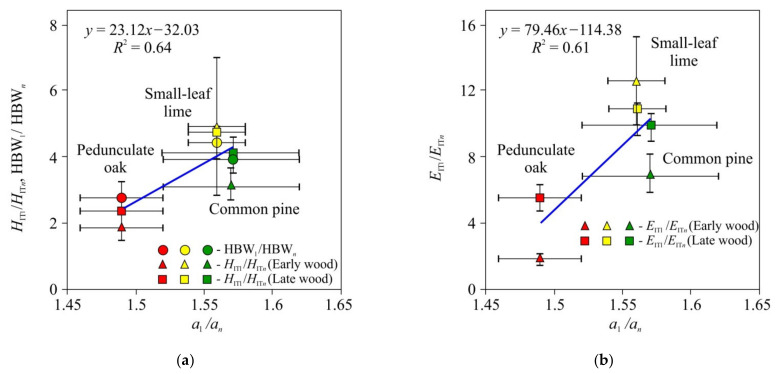
Dependencies of the physico-mechanical properties *H*_IT1_ /*H*_IT*n*_, HBW_1_/HBW*_n_* (**a**), and *E*_IT1_/*E_ITn_* (**b**) upon thermo-physical characteristics (*a*_1_/*a_n_*) for common pine*,* pedunculate oak, and small-leaf lime.

In the third stage of the experiments, tensor *a_ij_* components and Brinell hardness HBW were measured for the three wood species. Effective values of *H*_IT_, *E*_IT_, HBW, and thermal diffusivity anisotropy are presented in Table 1. As can be seen, all mechanical characteristics correlated with the ratio of the main components of the thermal diffusivity tensor, and for *E*, such correlation was about three times stronger than for *H_IT_* or HBW (Figure 9). It should be mentioned that the mechanical and thermal properties were correlated in all three studied species with significantly different microstructures.

## 4. Discussion

The thermal and mechanical properties of wood are determined, on both nano- and microscale, by the properties and nature of the interactions of their structural elements including cellulose nanocrystals, nano- and microfibrils, cell walls, and so on. As follows from general considerations, at the nanoscale, the higher the degree of crystallinity and ordering of cellulose molecules in nanofibrils, the lower the concentration of phonon scattering centers, so the higher the thermal conductivity, and at the same time, the higher the Young’s modulus and fibers’ strength. At the microscale, the higher the width of cell walls, the higher the wood density and the higher its thermal conductivity and strength. Therefore, one can expect a correlation between wood TPP and mechanical properties at least within the same wood species. Separately, these properties are well studied and generally follow the above-described basic concepts of heat transfer and irreversible deformation physics. For instance, thermal conductivity along the fibers is always higher than the transversal one [4,7]; the same is true for Young’s modulus and strength [8,10]. However, there is a lack of research on the thermal and mechanical properties of samples aimed and establishing a correlation between them while these properties vary significantly from sample to sample due to the uncontrolled nature of their establishment. This lack can be possibly ascribed to the highly laborious standard measurements of TPP and mechanical properties resulting from the necessity to cut a large number of samples, possibly contradicting restrictions on samples geometry, and to the destructive nature of mechanical testing. Current work utilizes non-destructive hardness and thermal diffusivity measurement techniques which do not require cutting the samples with a specific geometry. These techniques only require a flat region at the sample surface with an area of ~1 cm^2^. Besides, unlike the most traditional techniques [2,3,4,5,6,46,47], the thermal diffusivity measurement is contactless and requires just a few seconds. The relations between the longitude and transversal components of thermal diffusivity tensor (Figure 4b) obtained using this technique are in general in agreement with the ones obtained using various stationary and non-stationary methods such as extended dynamic plane source (EDPS) [2,3,47]. The ratio *a_l_/a_n_*—longitude to transversal thermal diffusivity—turned out to be more sensitive to wood moisture content than each *a_ii_* component alone. Therefore, it is more rational to use *a_l_/a_n_* as a TPP characteristics when searching for correlations between mechanical and thermal properties.

The study of the effective distribution of Young’s modulus and microhardness based upon indentations with size corresponding to that of several cells revealed that the mechanical properties differences between early and late wood annual ring layers (Figure 6, Figure 7 and Figure 8) were several times larger than those measured in cell walls at the nanoscale, which are usually around 10–20% [37,38,39]. Besides, the former technique require much less surface preparation and does not require a precise indentation targeting cell walls whose width is in the order of several micrometers. All of the above constitute a background for the development of new objective methods in dendrochronology and dendroclimatology with temporal resolution up to a week.

A parallel study of the mechanical and thermal properties of wood, cellulose-based materials, and other composites can lead to a better understanding of their nano- and microstructure and its role in determining the macroscopic properties, as well as in to the development of new techniques of contactless non-destructive express diagnostics. 

## 5. Conclusions

The dependency of Brinell hardness and thermal diffusivity tensor components upon humidity of common pine wood was demonstrated. Correlations between hardness and Young’s modulus measured both at microscopic and macroscopic levels on one hand and thermal diffusivity tensor components on the other hand are found. They were found both in single wood samples upon changing its humidity and across different wood species. Correlations between thermal diffusivity and Young’s modulus were more pronounced than between the former and hardness.

Analysis of the distribution of microhardness and Young’s modulus in the radial direction not only yielded the accurate location of growth rings boundaries and their width but also differentiated intra-annual wood layers. It could allow establishing relations between mechanical properties and tree growth conditions, not only on a year-to-year basis but also with within the growth season. Though accurate mapping of wood mechanical properties by means of nanoindentation is quite time-consuming, it can provide information with temporal resolution, which is not possible when using traditional techniques based on optical image analysis of growth rings structure. TPP measurements in a smaller regions and, hence, with better temporal resolution, are possible too by focusing a laser beam on smaller spots. Correlations between the mechanical properties and TPP help choose the method that better suits the testing goal. Such relations could be used in dendrochronology and dendroclimatology, complementing or substituting traditional methods based on optical properties variations by objective quantitative characterization of wood layers by mechanical properties distribution in addition to pure geometric parameters. As long as TPP measurements based on thermal imaging rely upon optical uniformity of the sample surface and TPP properties uniformity of the sample volume in the studied region, this method is also limited by the value of the ratio of the characteristic size of the heated region to the growth ring width and the width of its projection to the surface, which should be either several times lower or several times higher than the unity. 

Therefore, the obtained results imply that systematic studies could reveal reliable relations between humidity, hardness, and, possibly, strength on the one hand and thermal diffusivity on the other hand in wood of various species. Since the latter can be measured non-destructively and remotely using our express technique based on stepped local heating and thermal imaging, a perspective for the evaluation of wood mechanical characteristics by means of express evaluation of TPP is open.

## Figures and Tables

**Table 1:** Measured micro-/macromechanical and thermal physical properties of wood of the three studied species.

Type of Wood	Micromechanical Properties (Obtained by Nanoindentation with *P*_max_ = 500 mN)						Macromechanical Properties			TPP
	*E*_IT1_, GPa	*E*_IT*n*_, GPa	*E*_IT1_/*E*_IT*n*_	*H*_IT1_, MPa	*H*_IT*n*_, MPa	*H*_IT1_/*H*_IT*n*_	HBW_1_, MPa	HBW*_n_*, MPa	HBW_1_/HBW*_n_*	*a*_1_/*a_n_*
Common pine (*Pinus sylvestris* L.)										
Early wood	4.39 ± 0.04	0.44 ± 0.04	10 ± 1	57 ± 2	14 ± 1	4.0 ± 0.5	42 ± 2	11 ± 1	3.9 ± 0.7	1.57 ± 0.05
Late wood	12.4 ± 0.7	1.8 ± 0.2	7 ± 1	180 ± 10	58 ± 4	3.1 ± 0.4				
Pedunculate oak *(Quercus robur* L.)										
Early wood	3.1 ± 0.2	1.7 ± 0.2	1.9 ± 0.3	64 ± 3	34 ± 5	1.9 ± 0.4	67 ± 4	24 ± 3	2.8 ± 0.5	1.49 ± 0.03
Late wood	11.1 ± 0.8	2.0 ± 0.2	5.6 ± 0.8	190 ± 20	80 ± 8	2.4 ± 0.4				
Small-leaf Lime (*Tilia cordata* Mill.)										
Early wood	6.7 ± 0.1	0.61 ± 0.08	11 ± 2	75 ± 4	16 ± 3	5 ± 1	39.4 ± 1.5	8.9 ± 0.6	4.4 ± 0.5	1.56 ± 0.02
Late wood	8.6 ± 0.8	0.68 ± 0.08	13 ± 3	120 ± 20	24 ± 7	5 ± 2				

*E*_IT_—Indentation modulus, maximal load *P*_max_ = 500 mN; *H*_IT_—Indentation hardness, maximal load *P*_max_ = 500 mN; HBW—Brinell hardness (12.7 mm sphere diameter, 1 mm indentation depth); *a*—thermal diffusivity; Index 1—along the fibers; Index *n*—across the fibers.

## Data Availability

All the data is available within the manuscript.
